# A primary study of inverted right-angle configuration cannulated screws fixation of femoral neck fracture based on elaborated 3-dimensional features of the femoral head and neck region

**DOI:** 10.1371/journal.pone.0361871

**Published:** 2026-11-12

**Authors:** Changjian Liu, Hanwen Zhang, Yazhou Liu, Peihan Liu, Yang Hou

**Affiliations:** Department of Traumatology and Orthopedics, The First Hospital of Dalian Medical University, Dalian, China; University of Vigo, SPAIN

## Abstract

**Background:**

CT-based three-dimensional (3D) reconstruction was used to analyze the detailed anatomical morphology of the femoral head-neck region, and a modified inverted right-angle cannulated screw configuration was proposed to reduce the risk of posterosuperior screw in-out-in (IOI) placement during femoral neck fracture fixation.

**Methods:**

Imaging data from 22 patients (12 males and 10 females; mean age, 52.5 ± 16.8 years) were retrospectively analyzed. CT data of both hips were imported into Mimics to construct 3D digital femoral models for morphologic analysis and screw trajectory simulation. Based on the observed anterior offset of the femoral head-neck canal and the sloped posterosuperior cortex of the femoral neck, a modified inverted right-angle screw configuration and a negative anteversion fluoroscopic view were proposed. Technical feasibility was preliminarily illustrated in two patients with femoral neck fractures.

**Results:**

The femoral head-neck axis showed an anterior offset relative to the proximal femoral axis, which may increase the risk of posterosuperior IOI screw trajectory with the traditional inverted triangle configuration. In the 3D simulation, the modified inverted right-angle configuration placed the posterosuperior screw within the proposed safety zone. The negative anteversion fluoroscopic view appeared useful for identifying potential IOI screw trajectory. Technical feasibility of the proposed configuration was illustrated in two patients with femoral neck fractures, in whom no postoperative IOI screw displacement was observed on CT.

**Conclusion:**

The anatomical features of the femoral head-neck region may contribute to the risk of posterosuperior IOI screw trajectory with the traditional inverted triangle configuration. In this CT-based morphologic and simulation study, the modified inverted right-angle configuration showed a more favorable simulated position of the posterosuperior screw within the proposed safety zone, while negative anteversion fluoroscopy may help identify potential IOI screw trajectory. These preliminary findings require further validation through biomechanical studies and larger comparative clinical investigations.

## Introduction

Femoral neck fractures represent a common and severe type of musculoskeletal injury, accounting for approximately 3.58% of all systemic fractures and comprising about 54% of all hip fractures [1,2]. Despite the widespread use of internal fixation—particularly cannulated screw fixation—as a primary treatment modality [3], managing femoral neck fractures remains challenging due to the high incidence of postoperative complications, such as femoral head necrosis and nonunion, which significantly affect patient recovery and quality of life [4–7].

Cannulated screw fixation typically employs an inverted triangular configuration for screw placement [8–10]. Evidence suggests that the optimal distribution of screws, especially when positioned as close as to the femoral neck cortex, can enhance fixation stability and reduce the likelihood of postoperative complications [11–13]. However, the conventional inverted triangle configuration is not without drawbacks. Hoffmann et al.[14] reported that approximately 70% of posterior superior screws in this configuration exhibit an “in–out–in” (IOI) trajectory—penetrating the cortex of the posterior superior femoral neck, exiting, and re-entering the bone. This misplacement increases the risk of biomechanical failure and poor clinical outcomes [15].

To further validate this issue, Yuan et al. [11] conducted a study involving 107 patients with femoral neck fractures in 2020. Postoperative computed tomography (CT) scans revealed the IOI screw trajectory in 58% of patients, indicating that this complication is relatively prevalent and may substantially compromise prognosis. Importantly, intraoperative detection of this screw misplacement using conventional imaging techniques—such as anteroposterior, lateral, and modified lateral fluoroscopic views—is often unreliable [8], thereby increasing the likelihood of inadvertent IOI screw trajectory during surgery.

Given these concerns, the present study retrospectively analyzed imaging data from 22 patients with femoral neck fractures who were treated at the Department of Traumatology and Orthopedics, First Affiliated Hospital of Dalian Medical University, between September 2021 and March 2024. This study aimed to investigate the detailed anatomical morphology of the femoral head, neck, and proximal femur, and to explore strategies to avoid IOI screw trajectory of the posterosuperior screw during three-screw cannulated fixation. Although several modified screw configurations and fluoroscopic techniques have been proposed to reduce posterosuperior screw malposition and IOI phenomena, most remain based on the assumption that the femoral head-neck axis is collinear with the proximal femoral axis, and that conventional fluoroscopic views adequately represent posterior cortical anatomy. In contrast, our CT-based 3D analysis demonstrates a consistent anterior offset between the femoral head-neck medullary axis and the proximal femoral axis. Based on this anatomical finding, we propose a modified inverted right-angle configuration derived from axis mismatch rather than simple geometric redistribution of screws. In addition, we introduce a negative anteversion fluoroscopic view as a reverse-projection technique to better visualize the sloped posterosuperior cortex associated with IOI risk. Together, these approaches provide a different anatomical and imaging-based framework for understanding and potentially reducing posterosuperior IOI screw trajectory.

By analyzing femoral head-neck morphology and simulated screw trajectories, this study aimed to propose a modified screw configuration and an adjunctive fluoroscopic view that may help reduce the risk of posterosuperior IOI screw trajectory during femoral neck fracture fixation. The clinical applicability of this strategy was only preliminarily illustrated in two cases.

## Methods

### Study design and data collection

This study consisted of three components: (1) CT-based 3D morphologic analysis of the proximal femur, (2) simulation analysis of screw trajectories under different configurations, and (3) illustrative clinical feasibility cases.

The primary outcome was the morphologic characteristics of the femoral head-neck axis and posterosuperior cortical region. Secondary outcomes included simulation-based evaluation of screw trajectory patterns and feasibility assessment of the proposed configuration in illustrative cases.

This study presents a retrospective case analysis involving 22 patients diagnosed with femoral neck fractures. The clinical data were accessed for research purposes on 29/08/2024.. The inclusion criteria for this study encompassed patients who underwent CT imaging and met the predefined eligibility criteria.

CT scans and three-dimensional reconstruction data of the healthy femur were collected for these 22 individuals. Among the participants, 12 were male and 10 were female. The mean age was 52.5 ± 16.8 years, with an age range of 19–88 years. The mean height was 171.7 ± 6.9 cm, with a range of 158–181 cm. The mean weight was 68.0 ± 9.1 kg, ranging from 54 to 82 kg.

This retrospective study was approved by the Ethics Review Committee of the First Affiliated Hospital of Dalian Medical University (approval number: PJ-KS-KY-2024–534). The committee waived the requirement for written informed consent because of the retrospective nature of the study and the use of anonymized CT imaging data. Verbal informed consent was obtained from all participants before inclusion in the study. All CT data were anonymized before analysis, and no identifiable personal information was included in the study dataset.

### Study population

CT imaging data were retrospectively collected from adult patients who underwent lower-extremity CT examination at the First Affiliated Hospital of Dalian Medical University between September 2021 and March 2024. Participants were included if they were aged ≥18 years, had complete CT datasets with sufficient image quality for three-dimensional reconstruction, and showed no abnormalities affecting femoral morphology.

Exclusion criteria were: (1) previous surgery, fracture, or other trauma involving the femur or hip joint; (2) congenital or acquired deformities, developmental abnormalities, or pathological changes affecting femoral morphology; and (3) incomplete CT data or insufficient image quality for accurate reconstruction.

The participant was the sampling unit. For participants with bilateral morphologically normal femurs, one femur was selected according to a predefined protocol: the right femur was analyzed preferentially unless its image quality was inadequate, in which case the left femur was selected. Thus, the morphologic and simulation analyses included 22 femora from 22 participants (one femur per participant). Multiple cross-sections obtained within a femur were used to construct that femur's axes and minimum safety projection and were not treated as independent observations. The resulting participant-level measurements were used for the descriptive summaries. The two illustrative clinical cases were described separately and were not included as additional independent observations in the morphometric analysis.

### Measurement and analysis of the fine anatomical morphology of the femoral head neck and proximal femur

Application of Mimics 19.0 Software for CT Image Data Analysis: CT examinations were performed usiftng a dual-source photon-counting CT scanner (NAEOTOM Alpha, Siemens Healthineers, Forchheim, Germany). Images were reconstructed with a slice thickness and reconstruction interval of 1 mm. Despite reasonable efforts to retrieve the original imaging metadata, the exact manufacturer-specific reconstruction-kernel designation could not be identified from the archived imaging records. The DICOM data were imported into Mimics 19.0 software (Materialise, Leuven, Belgium) for three-dimensional femoral reconstruction.

Bone segmentation was performed using a CT attenuation threshold of 226–3071 HU, which was applied consistently across all datasets. Region growing, filling, and manual editing were used to refine the segmentation. Smoothing, cutting, and trimming were subsequently performed using a smoothing factor of 0.5 and one iteration of Laplacian smoothing to improve surface quality while preserving anatomical accuracy.

All segmentation, three-dimensional reconstruction, and quantitative measurements were independently performed by two experienced orthopedic investigators. Inter-observer reliability was evaluated using a two-way random-effects, absolute-agreement, single-measure intraclass correlation coefficient [ICC(A,1), equivalent to ICC (2,1)]. Excellent inter-observer reliability was observed for the femoral head-neck axis offset distance [ICC(A,1) = 0.95; 95% CI, 0.90–0.97], screw-distribution angle [ICC(A,1) = 0.92; 95% CI, 0.84–0.96], and posterosuperior cortical tilt angle [ICC(A,1) = 0.95; 95% CI, 0.88–0.98]. ICCs were calculated from the two investigators’ independent measurements before consensus resolution. After the reliability analysis, discrepancies were resolved by consensus, and the resulting consensus measurements were used in the descriptive analysis. Acquiring the Femoral Neck Axis and Proximal Femoral Medullary Cavity Axis: Starting from the base of the femoral neck and extending to the lower position of the femoral head, successive cuts were made at specified intervals, with the center points of the resulting segments being connected to form the axis. A vertical cross-section was taken just below the femoral neck, beginning from the lower end of the femoral head along the axis. Six evenly spaced cross-sections were made, and the largest inscribed circle within the medullary cavity was drawn. The centers of these six circles were then connected, forming a nearly straight line, which was defined as the femoral neck medullary cavity axis. Furthermore, at 3 mm intervals below the proximal lesser trochanter of the femur, six cross-sections were made, and five additional cross-sections were created. The largest inscribed circle within the medullary cavity was drawn for each, with the centers of these circles connected to form a nearly straight line, representing the proximal femoral medullary cavity axis (Fig 1).

**Fig 1:**
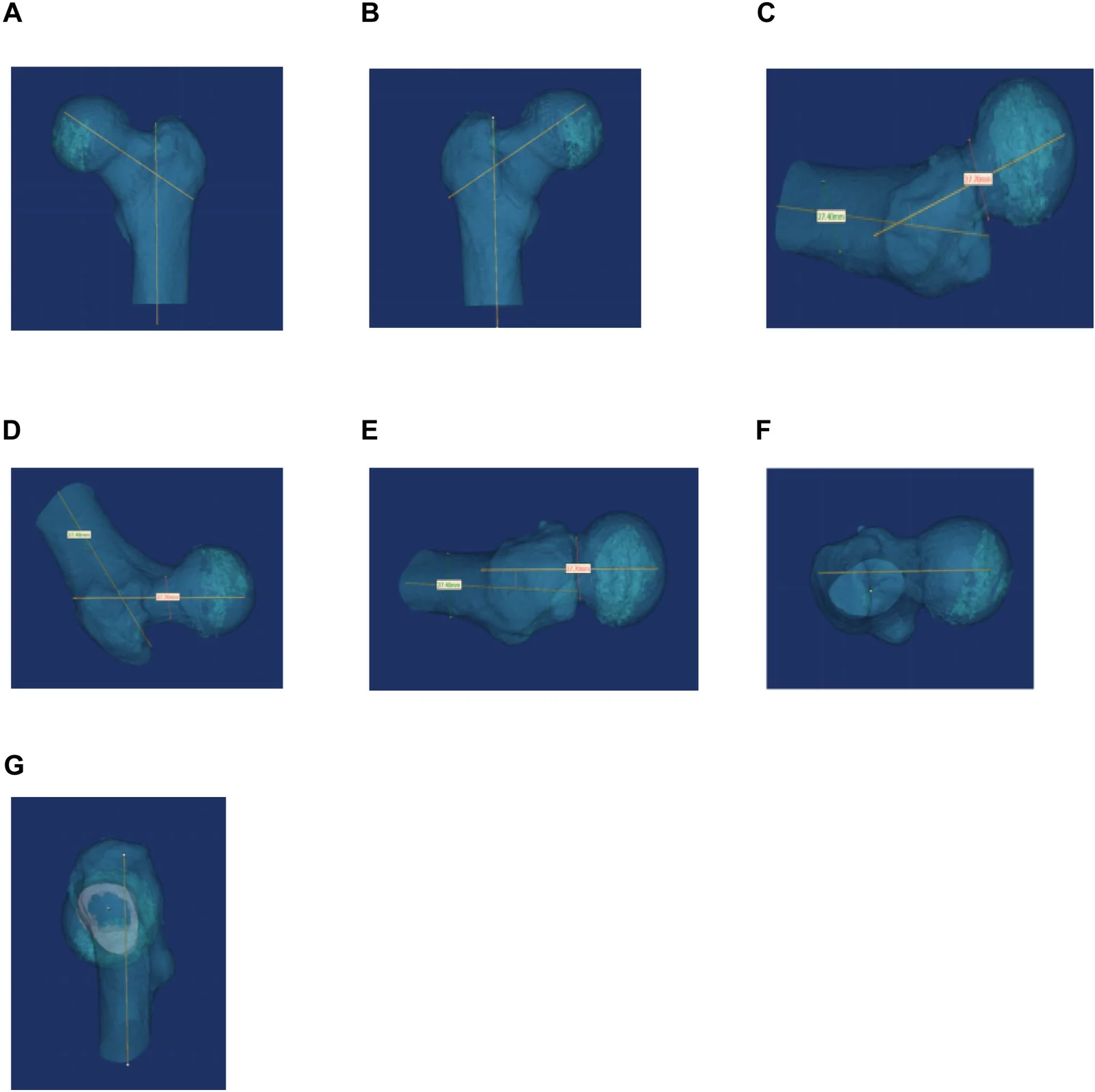
Establishment of the 3D femoral model and definition of the medullary axes. Representative model from the morphometric dataset (22 femora from 22 participants; one selected femur per participant). (A) Anteroposterior view with the femoral head-neck and proximal femoral medullary axes. (B) Posteroanterior view with the same axes. (C) Lateral view. (D) Negative anteversion view tangential to the posterosuperior femoral neck cortex. (E) Pure-lateral view without correction for femoral anteversion, showing anterior offset of the femoral head-neck axis relative to the proximal femoral axis. (F) Axial femoral-neck view showing the same offset. (G) Axial proximal-femoral view. The figure is illustrative; the reported offset distance was calculated at the femur level across all 22 analyzed femora (n = 22).

Initial Observation of the Relative Position Between the Femoral Head-Neck Axis and the Proximal Femoral Axis: The relative position of the femoral head-neck axis and the proximal femoral axis was initially observed. The 3D model was further used to measure the offset distance between the femoral head-neck medullary cavity axis and the proximal femoral medullary cavity axis in the lateral view. The morphology of the femoral head-neck medullary cavity and the best configuration of three cannulated screws at the narrowest point of the femoral neck axis were observed.

3D Model Simulation Analysis of Cannulated Screws in Traditional Inverted Triangle Configuration for Femoral Neck Fracture Fixation: This analysis simulated whether the screws would undergo IOI displacement after fixation of the femoral neck fracture with the traditional inverted triangle configuration. Using the femoral neck axis as the baseline, the direction of fixation of the femoral neck cannulated screws was determined. Simulations of anteroposterior (AP), lateral, and pure lateral (removing the forward tilt angle) views were performed. Three cannulated screws were placed in the traditional inverted triangle configuration to ensure the screws were as close as possible to the cortex, parallel to the axis, and distributed evenly for optimal fixation stability. Afterward, the different angles of views were assessed to determine whether the posterior screws cannulated experienced IOI displacement. The 3D data was used for multi-angle simulations to further confirm whether IOI occurred.

3D Model Simulation Analysis of the Best Distribution of Three Cannulated Screws to Prevent IOI Displacement: First, the multi-slice sections of the femoral neck were obtained, and the minimum safety projection section was determined. Perpendicular cuts to the femoral neck axis were made from the base to the lower position of the femoral neck, resulting in 6 segments. The 5 cross-sections of the 6 segments were projected along the femoral neck axis, and the overlapping section of all 5 projections represented the minimum safety projection plane of the femoral neck. Then, three cannulated screws were simulated to be inserted in the narrowest part of the femoral neck medullary cavity, ensuring the screws were as close as possible to the outer cortex and dispersed. Simulations of AP, lateral, and pure lateral views were performed to analyze whether

displacement occurred. Finally, 3D data with multi-angle simulations were used to assess if IOI truly occurred. The position of the posterior screws and the cause of IOI displacement after fixation using the traditional inverted triangle configuration were analyzed.

3D Model Measurement of the Femoral Neck Posterosuperior Cortex Tilt Angle: The tilt angle of the posterosuperior femoral neck cortical plane was measured using the plane of the posterosuperior femoral neck cortex and a direction perpendicular to the femoral neck axis. This simulated the “posterosuperior femoral neck tangential view” for visualization. The simulation was used to observe if IOI displacement occurred in the posterior screw after fixation of the femoral neck fracture with three cannulated screws.

### Illustrative clinical cases

Preliminary clinical feasibility cases. Two patients with femoral neck fractures who underwent fixation using the proposed technique were included as illustrative cases to assess the technical feasibility of the proposed configuration and fluoroscopic view. These cases were not intended for formal efficacy or safety evaluation. Case 1: A 51-year-old male patient presented with a Garden Type III femoral neck fracture resulting from a fall. The patient had a medical history of primary hypertension, diabetes mellitus, and renal insufficiency. The duration from injury to surgery was 4 days. Case 2: A 68-year-old female patient sustained a Garden Type II femoral neck fracture following a fall. The patient had a history of primary hypertension and diabetes mellitus. The time from injury to surgery was also 4 days.

Surgical Procedure: After completing the necessary preoperative preparations, both patients underwent femoral neck fracture closed reduction and internal fixation using three cannulated screws. The modified inverted right-angle cannulated screw fixation technique was employed during the procedure. To ensure accurate screw placement, fluoroscopic guidance was utilized, with negative anteversion of 30° and 34°.Postoperative Monitoring: Postoperative CT was performed to assess potential IOI displacement of the posterosuperior screw. The fracture healing process and any complications were carefully monitored and documented for both patients.

### Statistical analysis

Statistical analysis was descriptive. The participant was the sampling unit, and each participant contributed one selected femur; therefore, each reported morphometric summary was based on 22 independent femora from 22 participants (n = 22), unless otherwise specified. Multiple sectional observations within a femur were used only to derive femur-level axes or safety-zone geometry and were not entered as independent observations. Continuous variables were summarized as mean ± standard deviation (SD) and range, as appropriate, and distributions were assessed visually using histograms. No intergroup hypothesis testing was performed. The two illustrative clinical cases (n = 2 patients) were summarized separately and were excluded from the morphometric descriptive analysis. Analyses were performed using IBM SPSS Statistics version 25.0 and R version 4.3.1.

### Ethics approval and consent to participate

This retrospective study was approved by the Ethics Review Committee of the First Affiliated Hospital of Dalian Medical University (approval number: PJ-KS-KY-2024–534). The committee waived the requirement for written informed consent because of the retrospective nature of the study and the use of anonymized CT imaging data. Verbal informed consent was obtained from all participants before inclusion in the study

## Results

Anatomical Morphology of the Femoral Head-Neck and Proximal Femur: In the 22 analyzed femora from 22 participants, the three-dimensional (3D) models showed misalignment between the medullary axes of the femoral head-neck region and proximal femur. The femoral head-neck medullary axis was displaced anteriorly relative to the proximal femoral axis by a mean of 6.16 ± 0.86 mm (n = 22 femora).

Femoral Neck Medullary Cavity Morphology: Morphology was evaluated in 22 femora from 22 participants. The femoral neck medullary cavity became progressively irregular from the head toward the base (Fig 2). The overlapping axial projections defined an irregular minimum safety projection. In the 3D simulations, placement of three cannulated screws at this region yielded a modified inverted right-angle configuration rather than a conventional inverted triangle. The mean screw-distribution angle along the femoral neck axis was 89.76° ± 2.82° (n = 22 femora).

**Fig 2:**
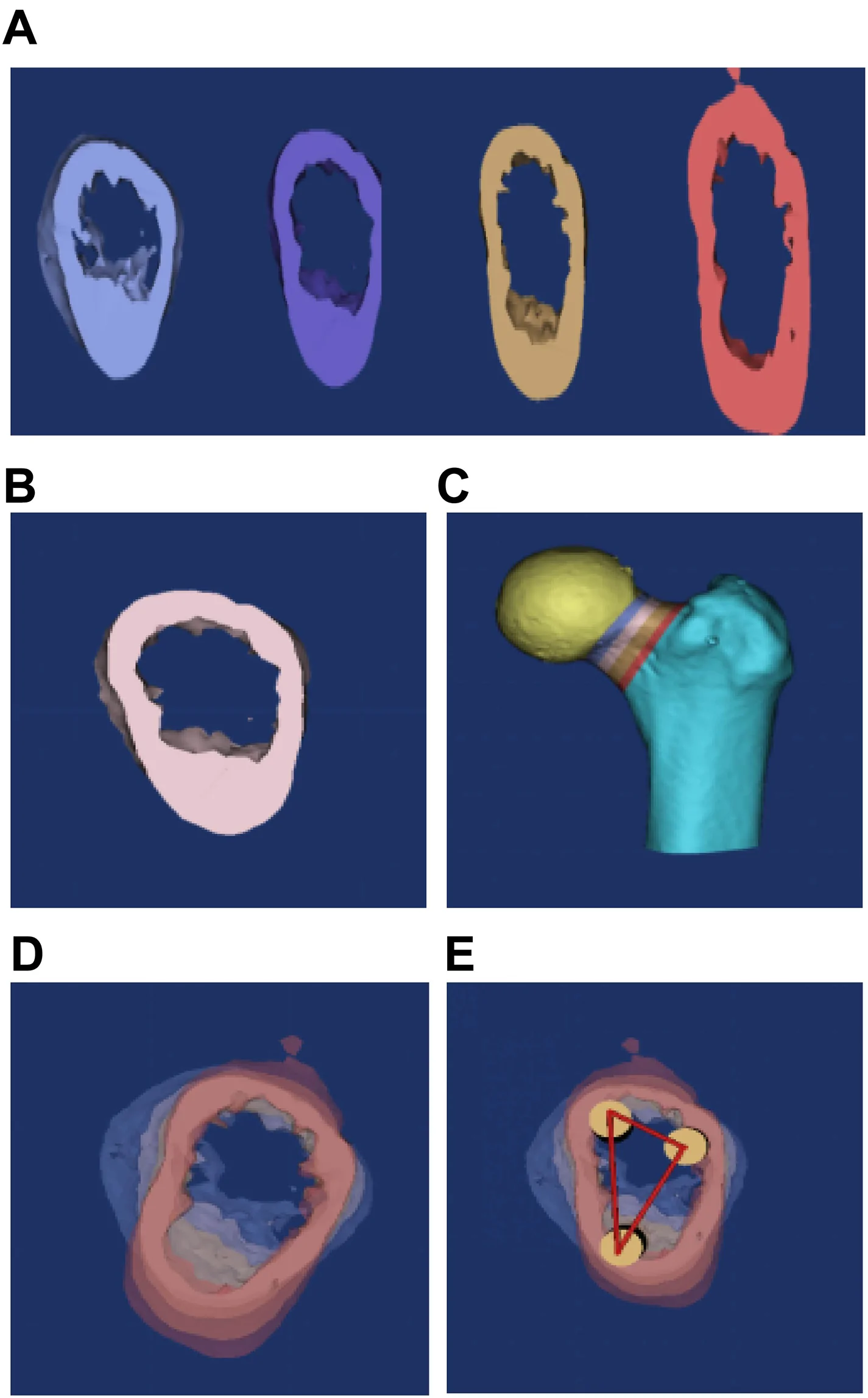
Construction of the femoral-neck minimum safety projection and simulated screw distribution. Representative 3D model from the morphometric dataset. (A) Serial axial cross-sections through the femoral-neck medullary canal. The light-blue, purple, tan, and red contours distinguish cross-sections obtained at different levels along the femoral neck; these colors are used only to register the section levels and do not represent different tissue types. (B) The pale-pink region represents the common overlap of the serial projections and defines the proposed minimum safety projection. (C) Locations of the serial sections on the 3D model: the yellow structure is the femoral head, the cyan structure is the femoral neck and proximal femur, and the purple, tan, and red bands mark representative section levels. (D) Semitransparent superimposition of the color-coded axial contours, illustrating the irregular medullary geometry and their common overlap. (E) Simulated modified inverted right-angle configuration within the minimum safety projection; the pale-yellow circles represent the cross-sections of the three simulated screws, and the red connecting lines show their spatial configuration. The figure is illustrative; the screw-distribution angle was summarized across all 22 analyzed femora (n = 22).

Traditional Inverted Triangle Configuration (Three Cannulated Screws): The traditional inverted-triangle trajectory was evaluated in 22 participant-specific femoral models (one femur per participant). In the representative model shown in Fig 3, the posterosuperior IOI trajectory was not apparent on anteroposterior, lateral, or pure-lateral projections but was demonstrated on the axial safety-zone view and negative-anteversion projection. Across the 22 femora, the mean posterosuperior cortical tilt angle used to define the negative-anteversion projection was 31.02° ± 3.32°.

**Fig 3:**
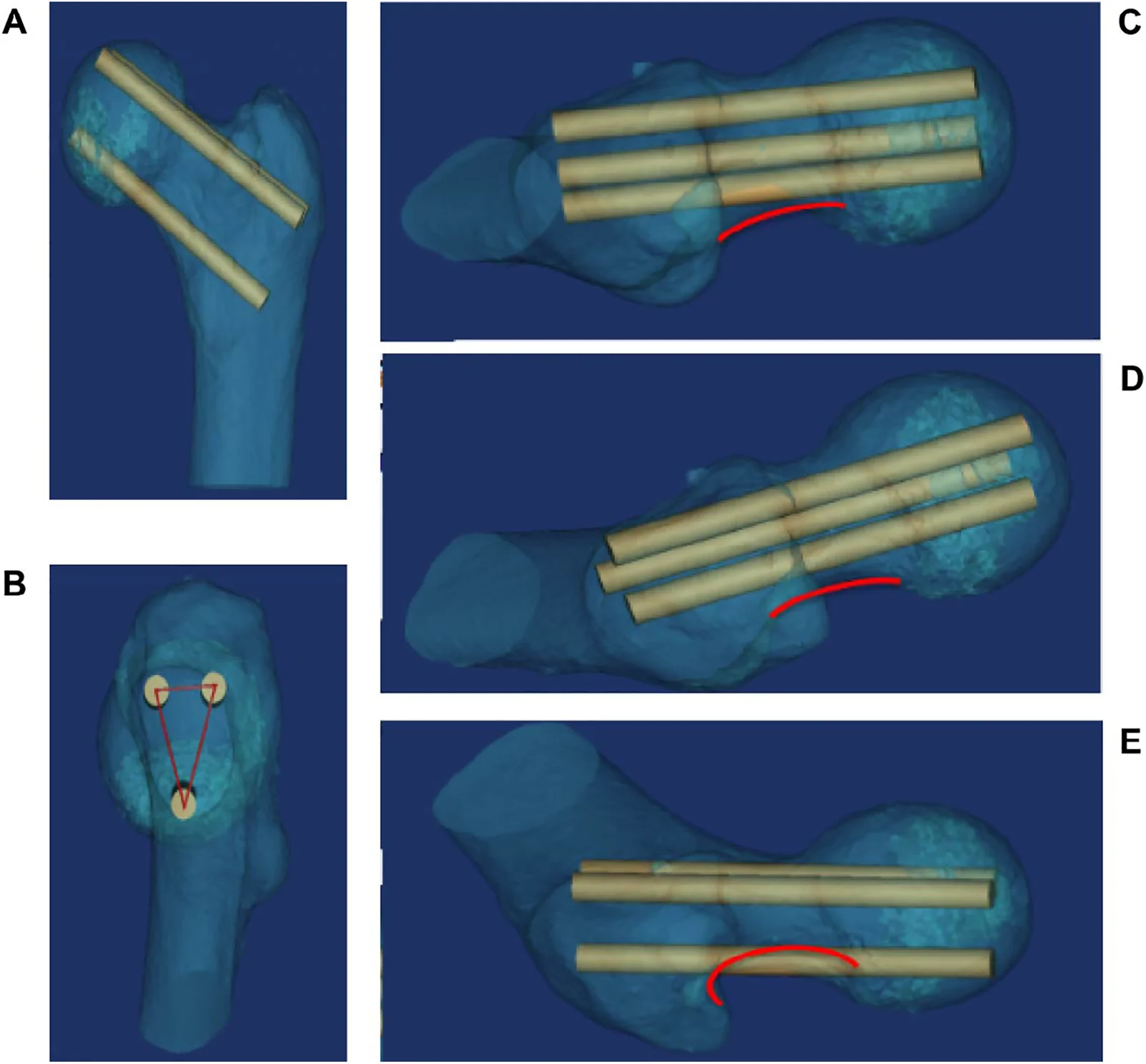
Traditional inverted-triangle screw simulation in a representative femoral model. The cyan surface represents the reconstructed proximal femur, and the beige cylinders represent the three simulated cannulated screws. (A) Anteroposterior view. (B) Axial view along the femoral-neck axis at the minimum safety projection; the pale-yellow circles represent screw cross-sections, and the red lines connect their centers to show the traditional inverted-triangle configuration. The posterosuperior screw crosses the proposed safety boundary and follows an in-out-in (IOI) trajectory. (C) Lateral view. (D) Pure-lateral view without correction for femoral anteversion. (E) Negative-anteversion tangential view of the posterosuperior femoral-neck cortex, demonstrating the IOI trajectory. In panels C-E, the red curved marking traces the projected posterosuperior cortical contour adjacent to the posterosuperior screw and provides a visual reference for assessing cortical violation. The IOI trajectory is not apparent on the anteroposterior, lateral, or pure-lateral views but is demonstrated on the axial and negative-anteversion views. This figure is an illustrative visualization; simulations were performed in 22 femora from 22 participants (one femur per participant; n = 22), with one trajectory set per configuration per femur.

Modified Inverted Right-Angle Configuration (Three Cannulated Screws): The modified configuration was simulated in the same 22 participant-specific femoral models. Fig 4 shows a representative model in which the posterosuperior screw remained within the proposed minimum safety projection on axial and negative-anteversion views. These simulations were descriptive; no inferential comparison or biomechanical test of superiority was performed.

**Fig 4:**
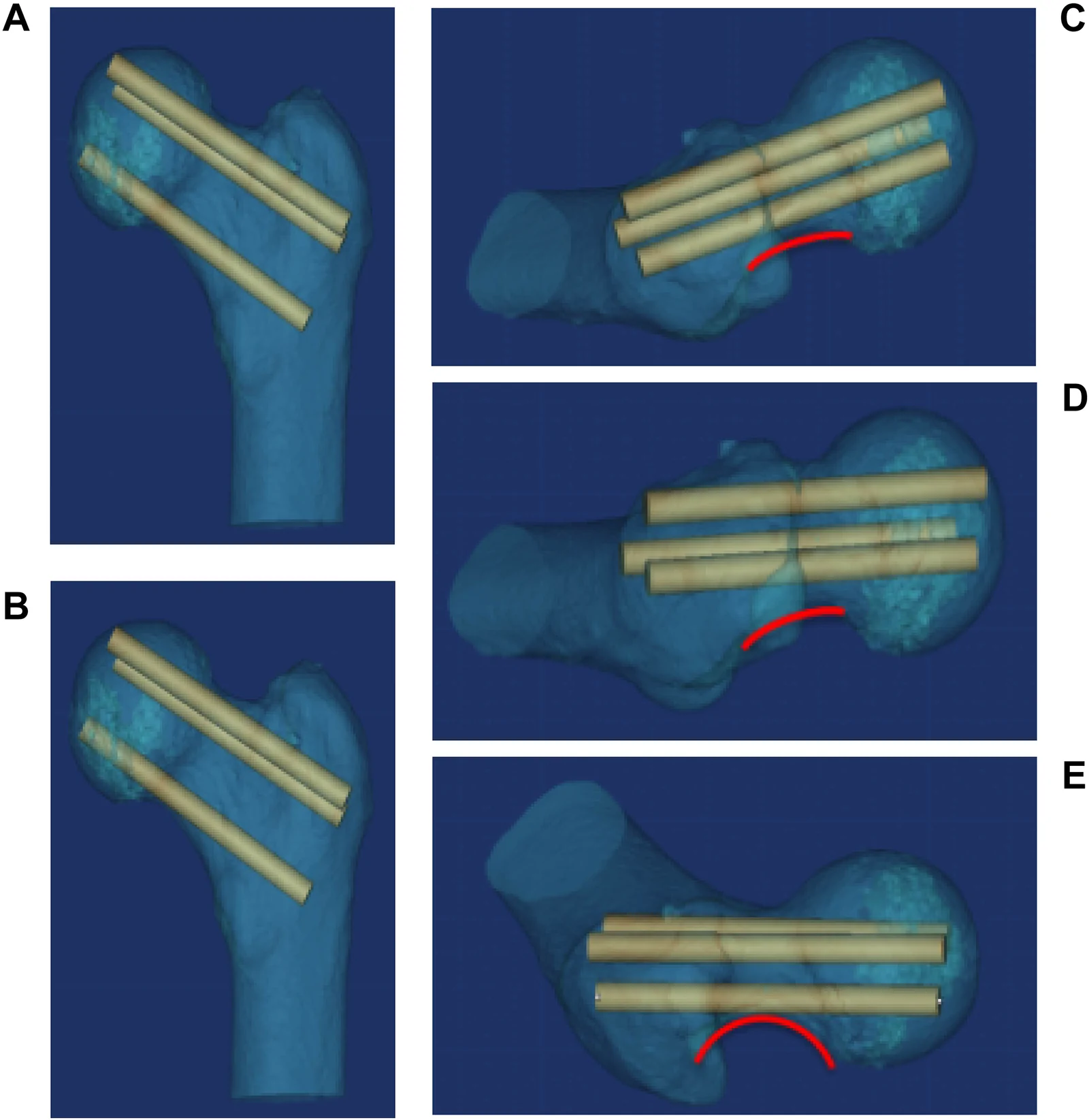
Modified inverted right-angle screw simulation in a representative femoral model. The cyan surface represents the reconstructed proximal femur, and the beige cylinders represent the three simulated cannulated screws. (A) Anteroposterior view. (B) Axial view along the femoral-neck axis at the minimum safety projection, showing the posterosuperior screw within the proposed safety boundary. (C) Lateral view. (D) Pure-lateral view without correction for femoral anteversion. (E) Negative-anteversion tangential view of the posterosuperior femoral-neck cortex, showing the posterosuperior screw within the proposed safety zone and close to the cortex. In panels C-E, the red curved marking traces the projected posterosuperior cortical contour adjacent to the posterosuperior screw and provides a visual reference for assessing screw clearance from the cortex. This figure is an illustrative visualization; simulations were performed in 22 femora from 22 participants (one femur per participant; n = 22), with one trajectory set per configuration per femur. No biomechanical or inferential comparison was performed.

Preliminary Clinical Feasibility Results: Two patients (n = 2; one treated hip per patient) underwent fixation using the proposed modified configuration under fluoroscopic guidance. Postoperative CT showed no IOI screw trajectory in either case. These observations were analyzed descriptively and separately from the 22-femur morphometric dataset and should not be interpreted as evidence of clinical effectiveness or safety.

Follow-up: The male patient was followed up at 4 months, while the female patient was followed up at 2 months. Both patients’ surgical incisions healed without incident, and no complications were noted. X-ray imaging confirmed the stability of the internal fixation with no signs of displacement. The male patient achieved bony union at 4 months post-surgery, whereas the female patient demonstrated partial fracture healing at 2 months. The female patient continues to be monitored and followed up.

Representative case images are presented in Fig 5. Because only two patients were included and the follow-up duration was limited, no conclusions can be drawn regarding complication rates, long-term healing, or superiority over conventional fixation.

**Fig 5:**
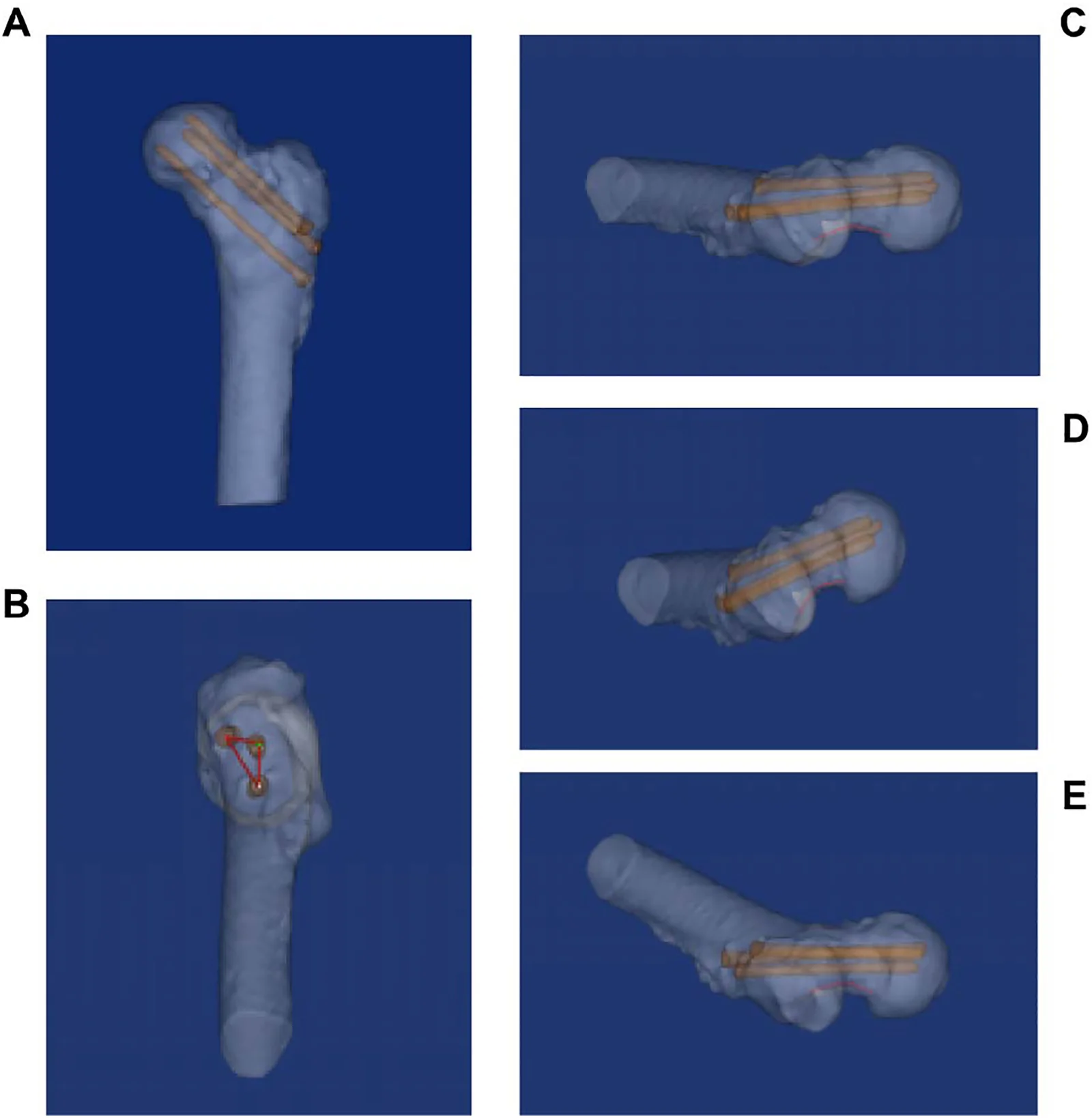
Postoperative CT-based 3D reconstruction of illustrative clinical case 1. Postoperative CT-derived 3D reconstructions obtained after internal fixation and before clinical follow-up in a 51-year-old man with a left femoral neck fracture treated using the modified inverted right-angle configuration (one patient and one treated hip; n = 1 for this figure). The translucent blue-gray surface represents the reconstructed proximal femur, the orange cylinders represent the three implanted cannulated screws, and the red curved marking identifies the posterosuperior cortical contour. (A) Anteroposterior view showing the overall screw configuration. (B) Axial femoral-neck view showing the three screw positions and their relationship to the cortical boundary. (C) Lateral view. (D) Pure-lateral view without correction for femoral anteversion. (E) Negative-anteversion tangential view used to assess the posterosuperior screw relative to the posterosuperior cortex. No posterosuperior IOI screw trajectory was identified on postoperative CT. Only case 1 is shown because this figure was intended as a representative technical illustration rather than a case-to-case comparison; case 2 was assessed separately and is described in the text but is not illustrated. Neither clinical case was included in the 22-femur morphometric descriptive analysis.

## Discussion

Femoral neck fractures represent a prevalent form of hip fracture. Due to the unique anatomical position of the femoral neck, the incidence of femoral head necrosis is relatively high, leading to poor prognosis and significant impairment of the patient's daily functioning. Conservative treatment may increase the risk of long-term complications. Currently, cannulates crew fixation is the dominant treatment approach, with the inverted triangle configuration regarded as the “gold standard” for femoral neck fracture fixation. It is recommended to place three screws to ensure optimal stability [15–18]. In recent years, studies have reported that intraoperative and postoperative fluoroscopic imaging demonstrates satisfactory screw positioning; however, a higher proportion of screws in the posterior superior region display the IOI screw trajectory postoperatively [19–21]. Hofmann et al [14]. conducted an in vitro specimen study, revealing that approximately 70% of the posterior superior screws in the traditional inverted triangle configuration exhibited the IOI screw trajectory. In 2020, Yuan et al [11]. reported on 107 cases of femoral neck fractures treated with cannulated screw fixation, where postoperative CT scans indicated that 58% of patients experienced the IOI screw trajectory. However, traditional intraoperative fluoroscopic views, including anteroposterior, lateral, and pure lateral (without anteversion) imaging, are insufficient for detecting the IOI screw trajectory [14,22]. Patients with femoral neck fractures often develop femoral head necrosis after internal fixation. When the posterior superior screw demonstrates the IOI screw trajectory, it can compromise the femoral head’s blood supply. This highlights the importance of clinicians paying close attention to the IOI screw trajectory [17,20,23,24].

This study, through three-dimensional CT reconstruction of the femoral head, neck, and proximal femur, revealed that the femoral head canal and the proximal femoral canal do not lie on the same plane [25,26]. Specifically, the femoral head canal is shifted approximately one-quarter anteriorly relative to the proximal femur. This anatomical variation increases the risk of the IOI screw trajectory in the posterior superior screw after cannulated screw fixation of the femoral neck using the traditional inverted triangle configuration. Surgeons may overlook this anatomical distinction and, assuming the femoral neck and proximal femoral canal are co-planar, may rely on palpating the lateral aspect of the greater trochanter as a reference for the proximal femoral canal to determine the anterior-posterior position of the cannulated screw entry point. This, in turn, may result in posterior screw placement, thereby heightening the risk of the IOI screw trajectory.

Additionally, the study observed that the posterior superior cortical surface of the femoral neck is irregular, slightly concave, and exhibits a sloped morphology, with the angle of the slope measured as 31.02° ± 3.32°. Moreover, the shape of the femoral neck canal is not the commonly assumed cylindrical form, but rather an irregular gradient. Therefore, using the minimum projection plane of the femoral neck axis as a reference for screw positioning may be a reasonable strategy to reduce the risk of posterosuperior IOI screw trajectory. However, this inference is based on morphologic analysis and simulation rather than direct biomechanical or comparative clinical evidence [27,28]. However, in actual surgical practice, it is challenging to obtain the minimum fluoroscopic section of the femoral neck axis. Zhang Yingze et al suggested that the morphology of the femoral neck influences screw fixation, recommending an adjustment of the traditional inverted triangle configuration by lowering the height of the posterior superior screw to reduce the potential for the IOI screw trajectory [29]. However, they did not specify the exact degree of reduction. In this study, the 3D model measured the morphology at the narrowest part of the axis canal, and the modified inverted right-angle configuration (89.73° ± 2.82°) showed a more favorable position of the posterosuperior screw within the proposed safety zone in the 3D simulation, while maintaining screw dispersion. Whether this translates into superior biomechanical performance or clinical outcomes remains to be determined. A deeper understanding of femoral neck morphology and anatomical characteristics may facilitate safer screw placement strategies and improve the accuracy of fixation.

Fluoroscopy remains the primary modality for monitoring the position of cannulated screws in femoral neck fracture fixation during surgery. The commonly employed fluoroscopic views include anteroposterior and lateral projections, typically facilitated by traction tables. With advancements in the understanding of femoral neck anatomy, recent years have seen the introduction of pure lateral fluoroscopy, which does not account for the anteversion angle. However, when utilizing the traditional inverted triangle configuration for cannulated screw fixation in femoral neck fractures, the IOI phenomena of screws often remain undetected in the anteroposterior, lateral, or pure lateral views, while CT scans frequently reveal its occurrence. This is closely related to our observation that “the femoral neck canal is shifted anteriorly by one-quarter compared to the proximal femur, and the femoral neck itself possesses an irregular quadrilateral structure with a slightly concave, sloped posterior superior cortex.”

In 2020, William et al reported a specialized fluoroscopic position to detect and correct the IOI screw trajectory of the posterior superior screw intraoperatively [30]. However, they did not systematically assess the fluoroscopic angles or the inclination angle of the posterior superior femoral neck surface.

In this study, CT data from 22 patients with normal femoral necks were analyzed to measure the anatomical characteristics of the proximal femur. The results showed that the inclination angle of the posterior superior cortex of the femoral neck was 31.02° ± 3.32°.

At this angle, tangential fluoroscopy may help identify potential posterosuperior IOI screw trajectory intraoperatively. Nevertheless, its practical value in routine surgery requires confirmation in larger prospective studies. Based on these preliminary findings, the additional negative anteversion fluoroscopic view may be considered as an adjunctive intraoperative view for detecting possible posterosuperior IOI screw trajectory. However, routine adoption should await further biomechanical and clinical validation.

Compared with previously reported modified screw configurations, which mainly adjust screw position or inclination within the conventional inverted triangular framework, the modified inverted right-angle configuration proposed in this study is based on a different anatomical premise. A consistent anterior offset between the femoral head-neck axis and the proximal femoral axis was identified, which is not considered in traditional configuration-based approaches. Similarly, most existing fluoroscopic techniques attempt to improve visualization through variations of standard AP, lateral, or oblique views. In contrast, the negative anteversion fluoroscopic concept introduced here is a reverse-projection approach derived from the posterosuperior cortical slope, rather than an empirically adjusted imaging angle. Overall, the present approach represents an anatomy-driven and projection-reversal framework rather than a simple modification of screw geometry or fluoroscopic positioning.

The technical feasibility of using the modified inverted right-angle configuration was preliminarily illustrated in two patients. However, because of the extremely limited sample size and short follow-up, no conclusions can be drawn regarding its safety, healing advantage, or clinical superiority over conventional techniques.

This finding is primarily based on 3D simulation analyses and two representative clinical cases without observed complications. However, given the relatively low incidence of this complication in clinical practice and the limited sample size, these findings should be interpreted as preliminary.

It is important to emphasize that the current evidence does not yet support changes to standard surgical practice. Further validation through larger prospective clinical studies, as well as biomechanical and cadaveric investigations, is required to confirm the reproducibility and clinical effectiveness of the proposed configuration.

An important point is that the present study does not provide direct comparative evidence against standard inverted triangle fixation, other modified screw configurations, or historical/matched clinical controls. Therefore, although the simulation findings suggest a potentially more favorable trajectory for the posterosuperior screw, no conclusion can be drawn regarding clinical superiority over existing fixation strategies. In addition, the current study did not include finite element analysis, cadaveric validation, or mechanical load testing. As such, any inference regarding fixation strength, mechanical stability, or resistance to failure would be premature. These issues should be addressed in future biomechanical and comparative clinical studies.

### Limitations

This study has several limitations. First, the sample size was small, and the clinical component included only two illustrative cases, which is insufficient to support conclusions regarding clinical effectiveness, safety, or improved outcomes. Second, the study was based mainly on CT morphologic analysis and screw trajectory simulation, without biomechanical testing, cadaveric validation, or comparative clinical controls. Third, the contralateral femur was used as a reference without formal assessment of bilateral symmetry. Fourth, no subgroup analysis was performed despite the wide age range of the included patients. Finally, long-term follow-up data were limited.

## Conclusions

The anatomical features of the femoral head-neck and proximal femur may contribute to posterosuperior IOI screw trajectory when the traditional inverted triangle configuration is used for femoral neck fracture fixation. In this CT-based morphologic and simulation study, the modified inverted right-angle configuration showed a more favorable simulated position of the posterosuperior screw, and negative anteversion fluoroscopy may help identify potential IOI placement intraoperatively. However, these findings remain preliminary and require further validation through biomechanical studies and larger comparative clinical investigations.

### Consent for publication

Written informed consent for publication of the clinical information and accompanying radiographic and CT images was obtained from both patients whose cases are presented in this manuscript.

## Abbreviations

3D: Three-dimensional
AP: Anteroposterior
CT: Computed tomography
DICOM: Digital Imaging and Communications in Medicine
FEA: Finite element analysis
HU: Hounsfield units
ICC: Intraclass correlation coefficient
IOI: In-out-in
LCP: Locking compression plate
SD: Standard deviation
